# Recombinant factor VIIa for uncontrollable bleeding in patients with extracorporeal membrane oxygenation: report on 15 cases and literature review

**DOI:** 10.1186/cc12581

**Published:** 2013-03-25

**Authors:** Xavier Repessé, Siu Ming Au, Nicolas Bréchot, Jean-Louis Trouillet, Pascal Leprince, Jean Chastre, Alain Combes, Charles-Edouard Luyt

**Affiliations:** 1 Service de Réanimation, Institut de Cardiologie, Groupe Hospitalier Pitié-Salpêtrière, Assistance Publique-Hôpitaux de Paris, 47, boulevard de l'Hôpital, 75651 Paris Cedex 13, France; 2 Service de Chirurgie Thoracique et Cardiovasculaire, Institut de Cardiologie, Groupe Hospitalier Pitié-Salpêtrière, Assistance Publique-Hôpitaux de Paris, 47, boulevard de l'Hôpital, 75651 Paris Cedex 13, France; 3 Université Paris 6-Pierre et Marie Curie, Sorbonne Universités, 4 place Jussieu, 75005 Paris, France

## Abstract

**Introduction:**

Bleeding is the most frequent complication in patients receiving venoarterial or venovenous extracorporeal membrane oxygenation (ECMO). Recombinant activated factor VII (rFVIIa) has been used in these patients with conflicting results. We describe our experience with rFVIIa for refractory bleeding in this setting and review the cases reported in the literature.

**Methods:**

Clinical characteristics, demographics, bleeding, thrombotic complications, mortality, and rFVIIa administration were retrospectively collected for analysis from the electronic charts of the 15 patients in our intensive care unit who received rFVIIa while being given ECMO from January 2006 to March 2011.

**Results:**

Fifteen patients received rFVIIa for persistent bleeding under venoarterial (*n *= 11) or venovenous (*n *= 4) ECMO. Bleeding dramatically decreased in 14 patients, without a major thrombotic event, except in one patient in whom a major stroke could not be ruled out. Two circuits were changed within the 48 hours after rFVIIa administration for clots in the membrane and decreased oxygenation but without massive clotting. The mortality rate was 60%.

**Conclusions:**

rFVIIa use for intractable hemorrhaging in patients receiving ECMO controlled bleeding, without major thrombotic events, and with 60% dying. Hence, its use warrants discussion, and clinicians should be aware of the possibility of potentially life-threatening systemic thrombosis, emboli, or circuit clotting. Whether rFVIIa can save the lives of such patients remains to be determined.

## Introduction

Extracorporeal membrane oxygenation (ECMO) may be used successfully in patients with refractory life-threatening cardiac or respiratory failure [1-3]. Venoarterial and venovenous extracorporeal devices require anticoagulant therapy to avoid thromboembolic complications, (for example, circuit thrombosis or pulmonary or systemic emboli) [2,4]. In addition, coagulation-cascade activation by the circuit and the membrane leads to platelet and coagulation-factor consumption, thereby increasing the risk of bleeding [5]. Recently, it was suggested that von Willebrand factor abnormalities induced by extracorporeal circulation could trigger acquired von Willebrand syndrome and could explain, at least in part, the elevated bleeding rate in these patients [6]. Indeed, hemorrhaging represents the most-frequent severe adverse event in patients receiving extracorporeal circulatory or respiratory support, with reported rates up to 30% [2,4]. Recombinant activated factor VII (rFVIIa; NovoSeven, Novo Nordisk, Copenhagen, Denmark) is a genetically engineered concentrate of human coagulation FVII that was originally designed to treat life-threatening hemorrhages in patients with hemophilia A or B [7]. To date, on-label indications of rFVIIa are prevention of bleeding for surgical interventions or bleeding episodes in patients with either congenital hemophilia A or B with antibody inhibitors against standard-factor replacements, or acquired hemophilia, or congenital factor VII deficiency. In recent years, its off-label use has increased in cardiac surgery [8-10], liver transplantation [11], or trauma [12] without affecting mortality rates in those settings [13,14]. rFVIIa has also been used successfully in infants and adults receiving ECMO [15,16]. However, circuit thrombosis, fatal or not, has been described for ECMO patients given rFVIIa with or without other prothrombotic drugs [17,18]. Because clotting of the circuit is the second most frequent complication after bleeding, the use of fVIIa may increase this risk and thus be more deleterious than beneficial.

Because data are scarce on rFVIIa use in patients with ECMO, we retrospectively reviewed 15 consecutive patients administered rFVIIa for life-threatening intractable bleeding while receiving ECMO and reviewed all the cases reported in the literature. Intractable bleeding was defined as persistent bleeding although all medical and surgical means to control bleeding have been exhausted.

## Materials and methods

### Patient selection and definitions

In our 18-bed tertiary intens ive care unit (ICU), ECMO is widely used for patients in refractory cardiogenic shock (venoarterial ECMO) or refractory acute respiratory distress syndrome (ARDS) (venovenous ECMO) [1,19,20]. Data on patients who received rFVIIa while receiving ECMO were extracted and retrospectively analyzed. Patients who underwent ECMO during their ICU stay but received rFVIIa before ECMO implantation or after its removal were not included. The following data were extracted from our computerized charts and recorded: demographic characteristics at ICU admission; ECMO type (venoarterial or venovenous), and reason for implantation; bleeding site; blood products required (number of units of packed red cells (PRCs), platelets and fresh-frozen plasma (FFP); specific medical, surgical, or interventional radiology hemostatic measures applied to stop bleeding; and details regarding rFVIIa use (total dose, number of infusions).

**Figure 1:**
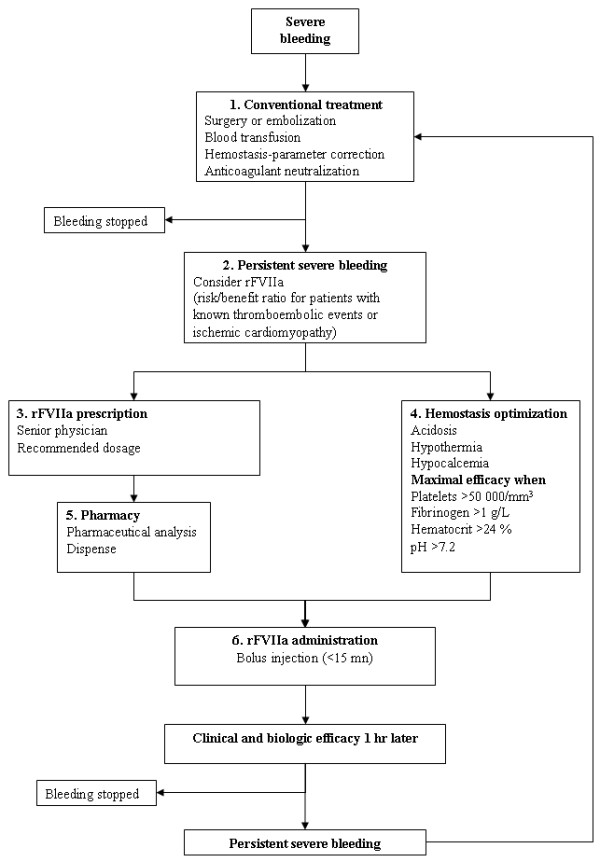
**Local guidelines for recombinant activated factor VII (rFVIIa) use to control refractory hemorrhage in patients with extracorporeal membrane oxygenation**.

In our institution, rFVIIa use adheres to guidelines recommending correction of coagulation parameters (prothrombin time (PT) > 50%, activated partial thromboplastin time (aPTT) ratio < 2, platelets > 50,000/ml, fibrinogen > 1 g/L), hypothermia (temperature > 36°C), hypocalcemia (calcemia > 2.2 m*M*), and anemia (hematocrit > 24%) before its infusion (Figure 1). Substitution limits for fibrinogen and platelets were 1 g/L and 50,000/ml, respectively. Our institution protocol for rFVIIa infusion is to give the patient an initial dose of 60 μg/kg, followed by a second dose of 60 μg/kg if the physician estimates that the bleeding does not stop. Factor XIII was never given, and we also never use antifibrinolytic agents such as tranexamic acid in such situations.

Number of blood products transfused (PRC, platelets, FFP, cryoprecipitate) and volume of chest-tube output (for patients with intrathoracic bleeding) were used to quantify bleeding. Volumes were recorded from 24 hours before until 24 hours after rFVIIa administration. In France, volumes of PRC, platelets, and FFP are 240 to 260 ml, 200 to 600 ml (depending on platelet concentration), and 200 to 220 ml, respectively. rFVIIa efficacy against bleeding was defined by its control (if visually in circuits), the diminished need for blood-product transfusion, and no early recurrence.

The following thrombotic complications were defined as clinically relevant thrombotic episodes: ischemic stroke or peripheral arterial embolism in patients with venoarterial ECMO, pulmonary embolism for patients on venovenous ECMO (suspected in the case of hemodynamic instability, unexplained oxygenation decrease), or circuit and/or oxygenator thrombosis for both ECMO types (either a total thrombosis requiring urgent changing of the circuit or partial thrombosis responsible for hemolysis). Duration of mechanical ventilation, ICU length of stay, and ICU mortality also were recorded.

This epidemiologic study did not require ethical approval, in accordance with the ethical standards of our institution's Committee for the Protection of Human Research Subjects (CCP Ile de France VI, Groupe Hospitalier Pitié-Salpêtrière, Paris). In accordance with French law, no informed consent was obtained because this epidemiologic study did not modify existing diagnostic or therapeutic strategies.

### ECMO circuit and implantation

The extracorporeal system consisted of polyvinyl chloride tubing, a membrane oxygenator (QuadroxBioline; Jostra-Maquet, Orléans, France; or Eos ECMO, Sorin, Milan, Italy), a centrifugal pump (Rotaflow, Jostra-Maquet, or Revolution, Sorin), and drainage and reinfusion cannulae (Biomedicus Carmeda, Medtronic, Boulogne-Billancourt, France, or Edwards Lifesciences, Irvine, CA, USA). An oxygen-air blender (Sechrist Industries, Anaheim, CA, USA) was used to ventilate the membrane oxygenator [1].

Two types of venoarterial ECMO were used: peripheral (femorofemoral) or central. In patients with peripheral ECMO, arterial and venous femoral cannulae were placed percutaneously or surgically. When the femoral artery caliber was small or signs of leg ischemia appeared, an additional 7Fr cannula was inserted distally into the femoral artery to prevent or treat severe leg ischemia [1]. Patients with central ECMO had right atrial drainage and aortic reinfusion cannulae.

For venovenous ECMO, two-site cannulation was used, and all cannulae were inserted percutaneously. The drainage cannula was inserted into the femoral vein (extending into the inferior vena cava), and the reinfusion cannula was inserted into the internal jugular vein (extending into the right atrium).

### Patient management under ECMO

All patients were continuously infused with unfractioned heparin to achieve an aPTT ratio < 1.5 for venovenous ECMO and between 1.5 and 2 for venoarterial ECMO. No heparin bolus was injected at ECMO initiation. The heparin dose was adapted at least once daily, according the aPTT value and clinical tolerance; it was stopped when bleeding occurred and restarted after bleeding was controlled. The oxygenator membrane was changed when fibrin deposition or thrombi had deleterious effects on blood oxygenation, the platelets count decreased significantly (< 50,000/ml), or hemolysis appeared. The circuit was checked daily by experienced perfusionists and changed when fibrin deposits or clots accumulated on the membrane, hemolysis or thrombopenia was observed, or blood oxygenation declined sharply, and systematically, every 8 to 10 days [1].

### Statistical analyses

Gaussian variables are expressed as mean ± SD, and non-gaussian variables, as medians (25^th ^to 75^th ^interquartile range (IQR)). Continuous variables were compared with the Student *t *test or the Mann-Whitney *U *test, as appropriate. Categoric variables were compared with the χ^2 ^test. All *P *values were two-tailed, and statistical significance was defined as *P *< 0.05. Analyses were performed by using StatView 5.0 (SAS Institute Inc., Cary, NC, USA) and SPSS 11.5 (SPSS Inc., Chicago, IL, USA) software.

## Results

### Study population

From January 2006 to March 2011, 315 of our ICU patients underwent venoarterial ECMO and, 62, venovenous ECMO. Among these 377 patients, 15 (4%) received rFVIIa while receiving ECMO. Four of them were supported with venovenous ECMO, and 11, 10 with preexisting cardiac disease, with venoarterial ECMO; their admission characteristics are listed in Table 1. Two patients were immunocompromised: an HIV-infected patient received venovenous ECMO, and an allogenic stem-cell transplantation recipient had venoarterial ECMO.

**Table 1:** Characteristics of the 15 patients

Characteristic	Value
Male sex	10 (66.7)
Age (years)	47 (32-53)
SAPS II at admission	79 (62-90)
SOFA at admission	15 (10-17)
McCabe & Jackson comorbidity score	0 (0-1)
< 2	12 (80)
≥ 2	3
Reason for ECMO	
Venoarterial ECMO	11 (73.3)
Postcardiotomy cardiogenic shock	5
Cardiac transplantation	1
Infarction-related cardiogenic shock	3
Myocarditis	2
Venovenous ECMO for ARDS	4 (26.7)
Pneumonia	3
Acute pancreatitis	1
Underlying disease	
Preexisting heart disease	10 (66.7)
Ischemic cardiomyopathy	4
Nonischemic dilated cardiomyopathy	2
Valvular cardiomyopathy	1
Hypertension-related cardiomyopathy	2
Congenital heart disease	1
Diabetes mellitus	1 (6.7)
Obesity (BMI > 30 kg/m^-2^)	3 (20)
30 < BMI < 35	2
35 < BMI < 40	1
BMI > 40	0
Immunocompromised	2 (13)
HIV infected	1
Allogenic stem-cell transplantation	1

Data are expressed as *n *(%) or median (IQR). ARDS, acute respiratory distress syndrome; BMI, body mass index; ECMO, extracorporeal membrane oxygenation; HIV, human immunodeficiency virus; SAPS, simplified acute physiology score; SOFA, sequential organ-failure assessment.

### Bleeding episode

For 12 (80%) patients (11 with venoarterial and one with venovenous ECMO), the source of bleeding was the surgical site (that is, mediastinal bleeding (all had a sternotomy, either for cardiac surgery or central ECMO cannulation)). The bleeding sources in the remaining three patients with venovenous ECMO are listed in Table 2. For all 15 patients, all medical and surgical means to control bleeding were exhausted before rFVIIa infusion: for the 12 patients with surgical sites, reinterventions excluded curable causes of bleeding; a patient with massive epistaxis received anterior and posterior nasopharyngeal packing; another with hemothorax required arterial embolization despite surgery because of persistent bleeding; and the last had uterine polyp-related hemorrhage treated with arterial embolization. These measures failed to control bleeding, as attested to by their transfusion requirements within the 24 hours preceding rFVIIa. Before rFVIIa injection, hemostasis parameters, body temperature, pH, and calcemia were within acceptable ranges recommended by our guidelines and the manufacturer for rFVIIa infusion. All patients received the same dosage for each rFVIIa infusion according to our institution guidelines, without protocol violation, (that is, a dose of 60 μg/kg for each infusion). Ten patients received one rFVIIa infusion, three received two, one required three, and the last was given four. Patients received a median rFVIIa dose of 77 (54 to 144) μg/kg.

**Table 2:** Bleeding characteristics and treatments administered to the 15 patients

Parameter	Value	
Site of bleeding		
Venoarterial ECMO		
Mediastinal (post-surgery)	11 (73.3)	
Venovenous ECMO		
Mediastinal (post-surgery)	1	
Hemothorax	1	
Gynecologic	1	
Epistaxis	1	
Transfusion required		
Packed red blood cell	17 (13.5-19)	
Fresh-frozen plasma	14 (9-21.5)	
Concentrated platelets	5 (3-8)	
Biologic parameters before and after rFVIIa infusion	Before	After
Hemoglobin	7.5 (7.1-8.6)	9 (8.2-9.8)
Platelets	83 (61-117)	68 (61-106)
PT	46 (42-52)	101 (54-120)
aPTT	1.7 (1.4-2.2)	1.4 (1.2-1.9)
Ionized calcemia	1.10 (1.05-1.20)	1.17 (1.03-1.28)
pH	7.37 (7.28-7.42)	7.41 (7.34-7.47)
Temperature before rFVIIa infusion (°C)	36.4 (34.3-37.1)	
rFVIIa dose infused (μg/kg)	77 (54-144)	
Number of injections of rFVIIa, number of patients		
1	10	
2	3	
3	1	
4	1	

Data are expressed as number (%) or medians (IQR). aPTT, activated thromboplastin time; ECMO, extracorporeal membrane oxygenation; PT, prothrombin time; rFVIIA, recombinant factor VIIa.

### Bleeding control

**Figure 2:**
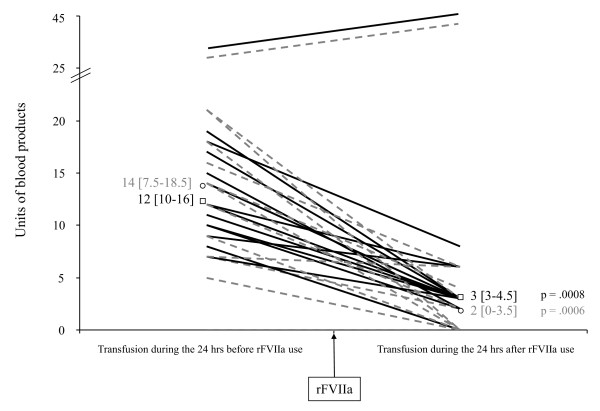
**Blood-product transfusions before and after recombinant activated factor VII (rFVIIa) infusion**. Each line represents one patient. Black lines, red blood cells; dashed gray line, fresh-frozen plasma, with respective medians [25^th ^to 75^th ^interquartile range] as □ and ○.

For 14 patients, bleeding dramatically decreased after rFVIIa infusion, as shown by the diminished need for blood products. Compared with the 24-hour period before rFVIIa infusion, RBC administration decreased a median of 75% (56.5% to 81%], and FFP declined 84% (67% to 100%) within the following 24 hours (*P *= 0.0008) (Figure 2). Moreover, for the 10 patients whose blood loss could be quantified, chest-tube output decreased from 2,057 (1,010 to 4,381) ml during the 24 hours before rFVIIa infusion to 850 (622 to 1,356] ml during the 24 hours after (*P *= 0.008). The patient whose bleeding persisted despite surgical intervention, arterial embolization, and rFVIIa infusion, underwent another surgical procedure; the surgeon found active bleeding of an injured intercostal artery that was finally controlled with vascular repair.

### rFVIIa-related thrombotic events

No clinical signs of thrombosis were observed after rFVIIa infusion. Two circuits in patients with venoarterial ECMO were changed within the 48 hours after rFVIIa infusion because of fibrin deposits and related hemolysis, but no massive circuit thrombosis occurred. One patient progressed to brain death during the 24 hours after the infusion, and because no brain CT scan was performed, an ischemic stroke could not be ruled out.

### Outcomes

The median durations of ECMO, mechanical ventilation, and ICU stay were 13 (6 to 37), 45 (6 to 49], and 54 (6 to 55) days, respectively. Nine (60%) of the 15 patients died, eight who had venoarterial ECMO; four developed multiorgan failure 1 (0 to 2) days after and despite controlling the hemorrhage; three patients died of septic shock 21 (14 to 88) days after bleeding stopped; two died of brain death within 24 hours after rFVIIa infusion, one of a cerebral hemorrhage (the possibility of an ischemic stroke with secondary conversion to a hemorrhagic stroke was ruled out by two experts, based on CT scan), and the other of unknown etiology. The only patient whose hemorrhage was not controlled by rFVIIa had active bleeding of an injured intercostal artery that was surgically repaired; he was discharged alive after > 100 days in the ICU.

## Discussion

Herein, we describe our experience with rFVIIa for refractory bleeding in 15 patients receiving ECMO. For 14 patients, bleeding dramatically decreased after rFVIIa infusion without relapse. Although no massive thrombotic events were observed in our patients, two ECMO circuits were changed during the 48 hours after rFVIIa administration because of oxygenator clotting that may have resulted from this procoagulant treatment. Those circuits were changed 8 and 11 days after ECMO initiation, which, in our experience, may be the normal lifetime of membranes. Moreover, one patient progressed to brain death early after rFVIIa infusion, and because ischemic stroke could not be excluded as its cause, a thrombotic complication could not be ruled out. However, another explanation in this patient could be a cerebral hemorrhage as the cause of death. If so, and despite the use of rFVIIa, the rate of cerebral hemorrhage in this population would be very high (13%). Although mortality was high, 40% of our patients with intractable bleeding survived. As a comparison, the survival rate of other ECMO patients (not having received rFVIIa) during the same time period in our unit was 55% (difference not statistically significant) [21].

To the best of our knowledge, we report the largest cohort of adult ECMO patients treated with rFVIIa. Several reports are available in the literature, nine of them focusing on pediatric patients (for a total of 48 infants) and six on adults (for a total of 9 adults) [15-18,22-30] (Table 3). All authors concurred about its control of bleeding and decrease of blood-products requirements. Some patients experienced circuit thromboses or oxygenator failure, and eight had systemic thromboses or emboli. Most events occurred in pediatric patients, and another procoagulant treatment was given in some cases. rFVIIa was given in doses ranging from 40 to 220 μg/kg. For the 26 patients for whom these data were available, eight who experienced thrombotic events had not received higher doses than had those without: 90 μg/kg (83 to 141) versus 192 μg/kg (93 to 252), respectively (*P *= 0.46). In our patients, except for one patient who progressed to brain death and in whom a major stroke could not be ruled out, no major thrombotic episode occurred. However, we did not measure thrombosis and fibrinolysis with thromboelastogram, D-dimer, or other tests. We thus cannot exclude that in some patients, the development of multiorgan failure may be related to, or have been favored by microthrombosis of end organs, and that this may have affected the mortality. Nevertheless, the lack of thrombotic complications in our patients contrasts with the high rate quoted in the literature. We can only speculate on the causes of this difference. We do not think it could be related to the dose of rFVIIa, because patients who experienced thrombotic events in the literature had not received higher doses than had ours. Interestingly, most thrombotic episodes occurred in pediatric patients: of the 16 patients who experienced a major thrombotic event, 14 were infants (30% of this population), and only two were adults (22% of this population). This higher rate in pediatric patients may be explained by differences in circuit size and by differences of clotting system between infants and adults.

**Table 3:** Studies reporting pediatric and adult patients received rFVIIa while receiving ECMO

Study	Number	Age, median (IQR)	rFVIIa dose (μg/kg)	rFVIIa doses received, number	Effect on blood-product requirements or bleeding	Thrombotic events	Deaths, number
Pediatric patients							
Wittenstein *et al. *[30]	4	0.625 (0.25-9) months	90-120	2	↓81.9% PRC between before and after one rFVIIa infusion ↓90% FFP between before and after one rFVIIa infusion	0	0
Velik-Salchner *et al. *[29]	1	0.5 months	90	1	Bleeding stopped	1 (arterial)	1
Dominguez *et al. *[24]	2	11 and 13 years	90	3 and 10	↓88.7% total blood products between 12 hours before and after rFVIIa infusion for case 1 ↓46.7% total blood products between 24 hours before and after rFVIIa infusion for case 2	0	1
Veldman *et al. *[16]	7	4 (0.4-14.5) months	83 (61-106)	3 (2-3.5)	↓23.1% PRC between before and after one rFVIIa infusion ↓35.3% FFP between before and after one rFVIIa infusion	0	3
Argawal *et al. *[22]	11^a^	9.5 days	36.5 ± 18.2	1.6 ± 7	↓42% PRC between before and after one rFVIIa infusion (*P *< 0.05) ↓59.3% FFP between before and after one rFVIIa infusion (*P *< .05)	2 circuit thromboses	3
Chalwin *et al. *[23]	1	18 months	90	1	Bleeding stopped	1 circuit thrombosis	1
Guzzeta *et al. *[26]	1	5 days	70	2	↓66.7% PRC after the two rFVIIa doses ↓100% FFP after two rFVIIa doses	1 extensive distal thrombotic emboli	1
Schneider *et al. *[28]	4^b^	6.7 (1-11.2) years	180 (146.25-180)	2 (1.75-2)	↓46.5% (-71.25 to -2.25) PRC between 12 hours before and after rFVIIa infusion 14.5% (-49.5 to 21.5) FFP between 24 hours before and after rFVIIa infusion	1 stroke	2
Niebler *et al. *[27]	17	0.1 (0-14) years	45-90	1.5 (1-2)	↓50% total blood products between before and after rFVIIa infusion (*P *= 0.05)	4 clinical thromboses 2 oxygenator failures 2 circuit obstructions	12
Adult patients							
Bui *et al. *[17]	1	56 years	146.5	2	↓53.8% PRC between the 12 hours before and after rFVIIa infusion ↓16.7% FFP between 12 hours before and after rFVIIa infusion	1 circuit thrombosis	1
Brose *et al. *[15]	1	17 years	220	2	Bleeding stopped (↓90% bleeding after rFVIIa infusion)	0	0
Schneider *et al. *[28]	5^b^	48 (45.2-52) years	90 (54-105)	2 (1-2)	↓61% (-79 to -47) PRC between the 12 hours before and after rFVIIa infusion ↓33% (-58 to 42) FFP between the 24 hours before and after injection of rFVIIa	0	3
Syburra *et al. *[35]	1	58 years	84.7	1	Bleeding stopped	1 left atrium thrombosis	1
Dunne *et al. *[25]	1	63 years	47	1	Bleeding stopped (↓90% bleeding after rFVIIa infusion)	0	0

FFP, fresh-frozen plasma; PRC, packed red cells; rFVIIa, recombinant activated factor VII. ^a^Analysis concerns 11 of the 12 patients on ECMO who received rFVIIa; one patient did not respond to rFVIIa. ^b^The authors reported on 12 patients, among whom four were infants with ECMO, five were adults with ECMO, and three were adults with ventricle-assist devices.

Many possibilities could explain bleeding in ECMO patients. First, surgeries before, after, or during ECMO are potential sites of bleeding. Second, even with percutaneous cannulation, the insertion site is another potential source of bleeding [2]. Third, coagulation disorders in patients on ECMO may be caused by heparin use, activation of platelets, and coagulation factors on the oxygenator membrane and/or specific coagulation-factor abnormalities. In addition to impaired platelet function, patients receiving ECMO may develop acquired von Willebrand syndrome [6]; shear stress during extracorporeal support is responsible for conformational modifications of von Willebrand factor that becomes exposed to metalloprotease-mediated cleavage [31]. The resulting truncated von Willebrand factor no longer has procoagulant activity [31]. A recent study by Heilmann *et al. *[6] clearly demonstrated that patients with ECMO developed acquired von Willebrand syndrome, whereas patients without any such support did not. Because of these coagulation disorders, the coagulation system should be closely monitored in patients with ECMO, to avoid bleeding but also thrombotic complications.

Logan *et al. *[32] recently evaluated off-label rFVIIa use in patients with intractable bleeding (2000 to 2008) and found that, in US hospitals, its use increased > 140-fold, so that in 2008, 97% of in-hospital administrations were off-label. Moreover, Yank *et al. *[14] evaluated the benefits and disadvantages of rFVIIa use for five off-label indications: intracranial hemorrhage, cardiac surgery, trauma, liver transplantation, and prostatectomy. They reported that, for intracranial hemorrhage, survival was not improved with rFVIIa use across a range of doses, but the arterial embolism rate increased; cardiac surgery-associated mortality was unchanged, but risk of thromboembolism was increased; and posttrauma mortality and thromboembolism rates were unaffected, but the ARDS risk was lower. Primary clotting disorders represented roughly 5% of off-label rFVIIa use in this recent analysis [14]. rFVIIa is an effective agent to treat refractory bleeding in von Willebrand disease patients and to treat or prevent bleeding in patients with alloantibodies or autoantibodies directed against von Willebrand factor [33]. According to a recent review, the positive response rate was 96%, with a low frequency of adverse events (only one case of myocardial infarction in a patient with type 2A von Willebrand disease) [34]. However, major thrombotic events can occur [35]. Thus, rFVIIa might be a potential therapeutic option for patients receiving ECMO with intractable bleeding despite conventional treatment (including normalization of coagulation parameters, surgery, and embolization), but clinicians must be aware of possible life-threatening systemic thrombosis, emboli, or circuit clotting.

Our study has several limitations. First, although it is the largest study to date, it is a small single-center study. However, the number of reported patients who received rFVIIa remains relatively low.

Second, because of its retrospective design, our study carries the inherent bias of this type of study. For example, bleeding control could be the natural evolution of the refractory bleeding episode due to coagulation optimization and/or interventional treatment rather than true rFVIIa efficacy. Nevertheless, our data on diminished blood-product needs and chest-drainage output clearly support rFVIIa efficacy, in agreement with the available literature.

Third, our study lacks a control group. Having a control group of ECMO patients with intractable bleeding but not treated with rFVIIa might have helped to determine whether the outcome would have been better with rFVIIa. However, because this disease is not frequent and because all patients with intractable bleeding received rFVIIa, this comparison is not possible.

Fourth, except for standard coagulation tests like aPTT or PT, we did not explore coagulation abnormalities more extensively, particularly in light of the recent description of acquired von Willebrand syndrome. Moreover, we did not measure von Willebrand antigen, ristocetin-cofactor activity, and collagen-binding capacity, known to be decreased in ECMO patients [6].

Fifth, rFVIIa use in ECMO patients remains off-label. Because of the scarcity of such cases, a randomized study to evaluate rFVIIa benefit in patients with persistent bleeding under ECMO is excluded. Although it seems, based on our patients and the literature, that rFVIIa may be useful in ECMO patients to stop bleeding, whether its use decreases mortality rate in this particular setting remains to be determined.

Last, because of our population, the results presented here apply mostly to bleeding in the setting of venoarterial ECMO and to surgical-site bleeding.

## Conclusions

In our experience, rFVIIa use for patients with ECMO with intractable bleeding was associated with bleeding control and decreased blood-product requirements. Except one patient who progressed to brain death and in whom a major stroke could not be ruled out, no major thrombotic episode occurred, but such events have been reported, mostly in pediatric patients, without any dose/effect relation. Despite a high mortality, 40% of our patients survived, similar to the previously published rate [15-18,22-30], and without statistical difference as compared with the 55% survival rate of our ECMO patients who did not receive rFVIIa. Therefore, we conclude that rFVIIa may be used to control refractory bleeding in patients receiving ECMO. However, its use should be considered, and clinicians should be aware of the risk of potentially life-threatening systemic thrombosis, emboli, or circuit clotting. Whether rFVIIa use in such patients may save lives remains to be determined.

## Key messages

• rFVIIa may be used to control intractable bleeding in ECMO patients.

• Before its use, clinicians should exhaust all medical and surgical means to control bleeding.

• Clinicians should be aware of the risk of major thrombotic events after rFVIIa.

• Whether rFVIIa may save lives in this population has not been determined.

• The coagulation system should be closely monitored in patients with ECMO to avoid bleeding and thrombotic complications.

## Abbreviations

aPTT: activated partial thromboplastin time; ARDS: acute respiratory distress syndrome; ECMO: extracorporeal membrane oxygenation; FFP: fresh-frozen plasma; ICU: intensive care unit; PT: prothrombin time; PRCs: packed red cells; rFVIIa: recombinant activated factor VII.

## Competing interests

The authors declare that they have no competing interests in relation to the present manuscript.

## Authors' contributions

XR, SMA, NB, JLT, PL, JC, AC, and CEL contributed to conception and design of the study. XR, SMA, NB, and CEL collected, analyzed, and interpreted the data. XR, JC, AC, and CEL drafted the manuscript. NB, JLT, and PL were involved in revising the manuscript critically for important intellectual content. All authors read and approved the final manuscript.
